# Standardization of three-dimensional pose of cylindrical implants from intraoral radiographs: a preliminary study

**DOI:** 10.1186/s12903-021-01448-9

**Published:** 2021-03-06

**Authors:** Saverio Cosola, Paolo Toti, Miguel Peñarrocha-Diago, Ugo Covani, Bruno Carlo Brevi, David Peñarrocha-Oltra

**Affiliations:** 1 Department of Stomatology, Tuscan Stomatological Institute, Foundation for Dental Clinic, Research and Continuing Education, Via Padre Ignazio da Carrara 39, 55042 Forte Dei Marmi, Italy; 2 Department of Multidisciplinary Regenerative Research, “Guglielmo Marconi University”, Via Plinio 44, 00193 Rome, Italy; 3 Department of Stomatology, Faculty of Medicine and Dentistry, University of ValenciaGascó, Oliag Street 1, 46010 Valencia, Spain; 4 Department of Maxillo-Facial Surgery (Acting Director: Dr. Bruno Brevi), Hospital and University of Pisa, Via Piero Trivella, 56124 Pisa, Italy

**Keywords:** Dental informatics/bioinformatic, Computer simulation, Dental implant(s), Digital imaging/radiology, Mathematical modeling

## Abstract

**Background:**

To introduce a theoretical solution to a posteriori describe the pose of a cylindrical dental fixture as appearing on radiographs; to experimentally validate the method described.

**Methods:**

The pose of a conventional dental implant was described by a triplet of angles (phi-pitch, theta-roll, and psi-yaw) which was calculated throughout vector analysis. Radiographic- and simulated-image obtained with an algorithm were compared to test effectiveness, reproducibility, and accuracy of the method. The length of the dental implant as appearing on the simulated image was calculated by the trigonometric function and then compared with real length as it appeared on a two-dimensional radiograph.

**Results:**

Twenty radiographs were analyzed for the present in silico and retrospective study. Among 40 fittings, 37 resulted as resolved with residuals ≤ 1 mm. Similar results were obtained for radiographic and simulated implants with absolute errors of − 1.1° ± 3.9° for phi; − 0.9° ± 4.1° for theta; 0° ± 1.1° for psi. The real and simulated length of the implants appeared to be heavily correlated. Linear dependence was verified by the results of the robust linear regression: 0.9757 (slope), + 0.1344 mm (intercept), and an adjusted coefficient of determination of 0.9054.

**Conclusions:**

The method allowed clinicians to calculate, a posteriori, a single real triplet of angles (phi, theta, psi) by analyzing a two-dimensional radiograph and to identify cases where standardization of repeated intraoral radiographies was not achieved. The a posteriori standardization of two-dimensional radiographs could allowed the clinicians to minimize the patient’s exposure to ionizing radiations for the measurement of marginal bone levels around dental implants.

## Background

Osseointegrated dental implants supporting fixed and removable prostheses were found to be highly effective rehabilitation strategies in the management of edentulous patients.

The radiographic assessment of the on-going peri-implant vertical bone loss after implant placement was an essential issue for clinicians to address [1].

Periapical and panoramic radiographs and computerized tomography allowed the clinician to measure marginal bone levels around a dental implant to evaluate its long-term stability, but with a varying degree of accuracy and reliability [1].

In two-dimensional radiography, different types of intraoral and extraoral equipment met the requirements for standardized evaluation of peri-implant marginal bone loss [2]. These types of equipment took into account the analytic basis of projective geometry which consisted of specific features used for describing the spatial relationships between x-ray source and object, and the complex relationships existing between the various layers (object plane image and detector plane image) [3].

The determination of the three-dimensional position and orientation of known objects from single perspective projections were initially proposed by Hoffmann and Esthappan with a priori knowledge of positions of the specific markers [4]. Generally, external markers (alone or in combination with a holding device) were and are employed to allow for an accurate estimate a posteriori of the object’s position and orientation, thereby providing both the referral points in space (set of three radiopaque ball bearings or spheres) and the known dimensions of the object being analyzed [5, 6].

Others suggested the possibilities of estimating fixture angulation, dimension, and position through distortion on its shadow as it appeared in the projective plane [7–11].

In computer vision, the pose of an object was the combination of position and orientation relative to some external and independent coordinate system. Here, pose referred to the orientation of the cylindrical implant concerning the image-coordinate frame. The “within-object features” was a direct consequence of the abovementioned footsteps, proposing to use the dental implant as a reference object of known dimensions in the image without any external markers [12]. That's was made to minimize information about the position and to focus solely on the orientation. The procedure consisted of making the most of the more complex radiographic shadow of a conventional dental implant, which had, at least in part, the shape of a right circular cylinder with known diameter, brought.

In previous studies, rotational poses of the nonconventional implants, with or without effects of image distortion, had been described; the components of the lengthwise- and crosswise-vector allowed clinicians to estimate sets of angles describing the position of an implant based on its appearance on a single two-dimensional radiograph [13].

However, the abovementioned methods worked only with a non-cylindrical object, such as a blade implant, in which lengthwise- and crosswise-vector were orthogonal to one another and easy to find using an implant projection. At first glance, it might seem that rotation of the implant around its main axis does not provide meaningful information since the implant had rotational symmetry. Rotating the implant about the long axis did not roughly influence its radiographic shadow but any degree turn about this axis resulted in a rotated image projection of the bone surrounding the implant by the same degree. So the calculus of this angle from a radiographic shadow was extremely important to ensure consistent reproducibility in marginal bone assessment, which was measured along the line of the implant.

The meaning of the paper was that the authors took patient radiographs with implant-images (implant dimensions were known from CT or optical scans) and calculated their “pose” relative to a referring point using an algorithm [3]. Then the authors simulated this position again using another algorithm [3] and compared computed differences between real measurements and the ones from the simulations.

The present article will:

1. introduce a theoretical solution to obtain a triplet of angles (φ, θ, and ψ) describing the pose of the cylindrical fixture as shown, a posteriori, in a single two-dimensional radiograph;
2. present in silico and experimental application of the method for accuracy;
3. verify the effectiveness of the method, attempting to relate real-length data to those calculated by the angular correction factor simulation.

## Methods

### Theory

Especially in the measurement of the marginal bone level around dental implants in repeated intraoral radiographs of the same patient, a very similar acquisition process, for which it's hard to quantify the number of errors attributed to a series of characteristics, was needed. To properly fulfill standardized two-dimensional periapical radiography of a cylindrical dental implant in the alveolar bone, the following general requirements had to be respected:

- Three-dimensional position: angulations and translations in three-dimensional space of the dental implant as equal to each other as possible could be achieved with a customized bite block often formed of a rigid material combined with an extension cone paralleling device (an XCP-ORA, the eXtention Cone Paralleling-One Ring and Arm positioning system, Dentsply International, Elgin, Illinois, USA);
- Magnification: an image’s size could be either enlarged or reduced depending on the distance from the x-ray source to the detector, that is directly proportional to the length of the XCP-ORA’s arm;
- Distortion: it could arise from an angle between the implant longitudinal axis and the imaging plane or a very oblique projection. An error in the misalignment could most likely be generated because the Indicating Device (PID) of the Dental X-ray Tube (DXT) head was manually and poorly placed by the clinician without the help of the XCP device or because the patient moved during the measurement.

**Fig. 1:**
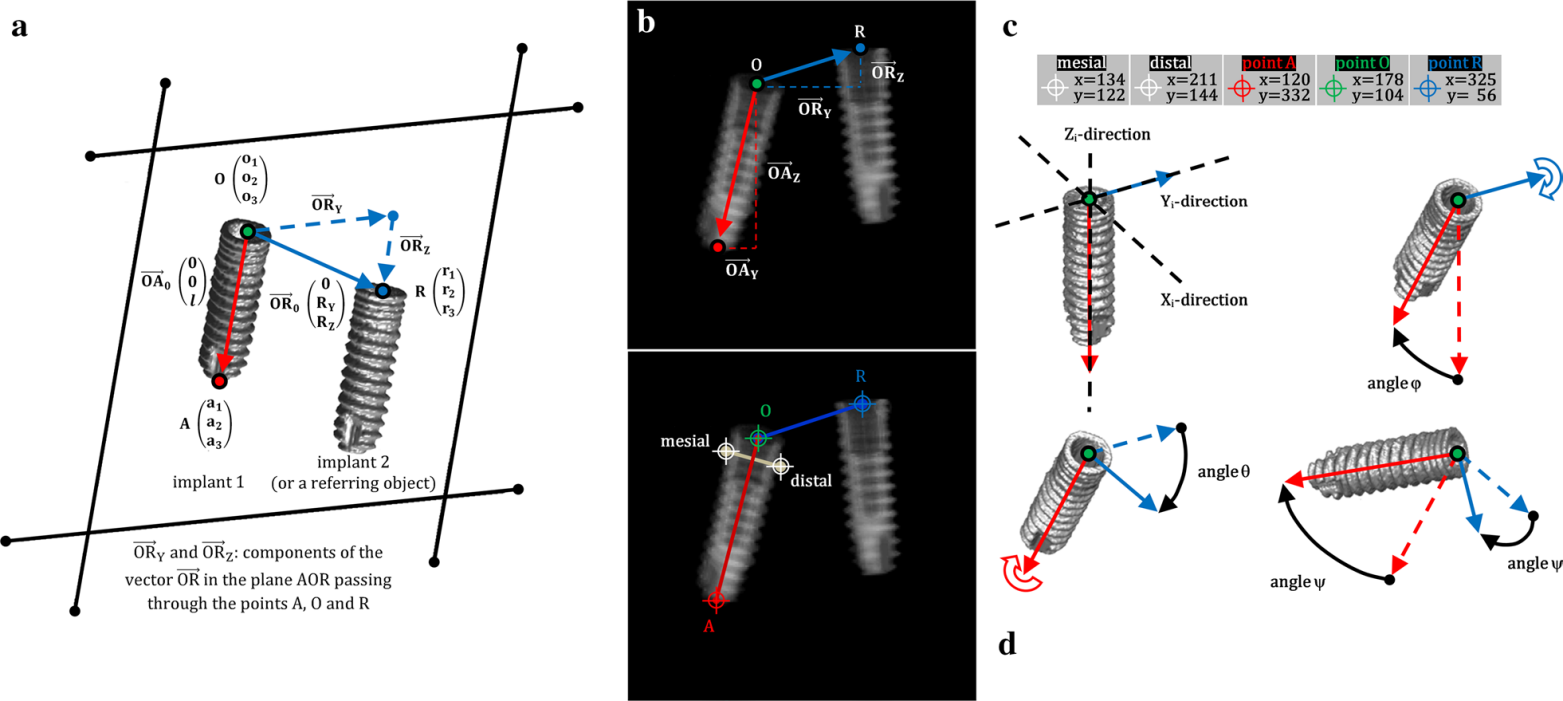
**a** Line from the neck of the cylindrical implant to the apex, and symmetrical from left to right, is the direction $\overrightarrow{\mathrm{OA}}$; the direction $\overrightarrow{\mathrm{OA}}$ touches the centre of the implant shoulder in the implant reference point, i.e., the point O. Line from the point O to the external reference point, i.e., the point R, is the direction $\overrightarrow{\mathrm{OR}}$; in present example the point R is the centre of the implant shoulder of a further dental implant. ${\overrightarrow{\mathrm{OA}}}_{0}$ = (0 0 l) were the lengthwise starting vector (red arrow), where *l* is the implant length, and R_Y_ and R_Z_ are the components of the crosswise vector (blue arrow), ${\overrightarrow{\mathrm{OR}}}_{0}$ = (0 R_Y_ R_Z_), along plane AOR, represented by the trapezoid indicated by the thin black lines, and passing through point A = (a_1_, a_2_, a_3_), O = (o_1_, o_2_, o_3_) and R = (r_1_, r_2_, r_3_). **b** Drawing of the two vectors, $\overrightarrow{\mathrm{OA}}$ and $\overrightarrow{\mathrm{OR}}$ on mega pixel simulated two-dimensional image of a dental implant with measured reference points on another implant: mesial point, distal point, point A, point O and point R with (**c**) the list of variables and that of their values as obtained from the free standalone software Osiris 4.19 which was used to acquire the two-dimensional coordinates of the points. **d** Three-dimensional renderings of the cylindrical implant using the three consecutive rotational angles φ, θ and ψ along the three direction of the main implant axes X_i_, Y_i_ and Z_i_

The effects of both the image magnification level and the relative translation between dental implant and frame of the image were not significant because the dental implant itself was the object of the investigation and acted as a benchmark for further analyses. After the components related to translation and magnification had been deleted, just three rotation components might be determined for describing implant orientation. The simplest way to do it is to describe the initial position of the cylindrical dental implant, given by the position of the apices described as the vector *v*_0_ = (x_0_, y_0_, z_0_). which is perpendicular to the ground (that is, perpendicular to the XY-plane along the implant direction Z_i_ and parallel to both the XZ- and YZ-plane, with implant directions Y_i_ and X_i_, respectively as appearing in Fig. 1). After applying a rotational matrix to the vector, its final position will be *v*_1_ = (x_1_, y_1_, z_1_) which sets the viewpoint after the rotational-steps have been completed with three angles of rotation, pitch-φ, roll-θ, and yaw-ψ, those correspond to three separate and successive rotations along the implant main direction X_i_, Z_i_, and Y_i_.

The vector is rotated in space and projected on the detector plane (YZ-plane) as described by the following components which depended on the triad of angles (φ, θ, ψ) and the magnitude of the vector’s components. For each main axis a Rodrigues rotational matrix, ${R}_{\widehat{\omega }}\left(\alpha \right)={e}^{\widehat{\omega }\alpha }$ corresponding to a rotation by an angle α about a fixed axis specified by the unit vector $\widehat{\omega }$ with the following explicit formula ${R}_{\widehat{\omega }}\left(\alpha \right)=\widehat{i}+\widehat{\omega }\mathrm{sin}\alpha +{\widehat{\omega }}^{2}\left(1-\mathrm{cos}\alpha \right)$ has been applied. T_1_ is the rotational matrix obtained by rotating the implant along the Y_i_-direction and angle φ with its explicit form from Rodrigues’s second equation:

1 $$\begin{aligned} {\text{T}}_{1} & = \left( {\begin{array}{*{20}c} 1 & 0 & 0 \\ 0 & 1 & 0 \\ 0 & 0 & 1 \\ \end{array} } \right) + \left( {\begin{array}{*{20}c} 0 & 0 & 1 \\ 0 & 0 & 0 \\ { - 1} & 0 & 0 \\ \end{array} } \right)\sin \upvarphi \\ & \quad + \left( {\begin{array}{*{20}c} 0 & 0 & 1 \\ 0 & 0 & 0 \\ { - 1} & 0 & 0 \\ \end{array} } \right)^{2} \left( {1 - \cos \upvarphi } \right) \\ \end{aligned}$$

2 $${\text{T}}_{1} = \left( {\begin{array}{*{20}c} {\cos \upvarphi } & 0 & {\sin \upvarphi } \\ 0 & 1 & 0 \\ { - \sin \upvarphi } & 0 & {\cos \upvarphi } \\ \end{array} } \right)$$

T_2_ is the second rotational matrix obtained by rotating the implant along the Z_i_-direction (after rotation of φ) with angle θ:

3 $$\begin{aligned} {\text{T}}_{2} & = \left( {\begin{array}{*{20}c} 1 & 0 & 0 \\ 0 & 1 & 0 \\ 0 & 0 & 1 \\ \end{array} } \right) + \left( {\begin{array}{*{20}c} 0 & {\cos \upvarphi } & 0 \\ { - \cos \upvarphi } & 0 & { - \sin \upvarphi } \\ 0 & {\sin \upvarphi } & 0 \\ \end{array} } \right) \sin \uptheta \\ & \quad - \left( {\begin{array}{*{20}c} 0 & {\cos \upvarphi } & 0 \\ { - \cos \upvarphi } & 0 & { - \sin \upvarphi } \\ 0 & {\sin \upvarphi } & 0 \\ \end{array} } \right)^{2} \left( {1 - \cos \uptheta } \right) \\ \end{aligned}$$

4 $${\text{T}}_{2} = \left( {\begin{array}{*{20}c} {\cos \uptheta + \sin^{2} \upvarphi - \sin^{2} \upvarphi \cos \uptheta } & {\cos \upvarphi \sin \uptheta } & { - \sin \upvarphi \cos \upvarphi + \sin \upvarphi \cos \upvarphi \cos \uptheta } \\ { - \cos \upvarphi \sin \uptheta } & {\cos \uptheta } & { - \sin \upvarphi \sin \uptheta } \\ { - \sin \upvarphi \cos \upvarphi + \sin \upvarphi \cos \upvarphi \cos \uptheta } & {\sin \upvarphi \sin \uptheta } & {\cos \uptheta + \cos^{2} \upvarphi - \cos^{2} \upvarphi \cos \uptheta } \\ \end{array} } \right)$$

T_3_ is the third rotational matrix obtained by rotating the implant along the X_i_-direction (after φ and θ rotations) with angle ψ:

5 $$\begin{aligned} {\text{T}}_{3} & = \left( {\begin{array}{*{20}c} 1 & 0 & 0 \\ 0 & 1 & 0 \\ 0 & 0 & 1 \\ \end{array} } \right) + \left( {\begin{array}{*{20}c} 0 & { - \sin \upvarphi \cos \uptheta } & {\sin \uptheta } \\ {\sin \upvarphi \cos \uptheta } & 0 & { - \cos \upvarphi \cos \uptheta } \\ { - \sin \uptheta } & {\cos \upvarphi \cos \uptheta } & 0 \\ \end{array} } \right) \sin \uppsi \\ & \quad - \left( {\begin{array}{*{20}c} 0 & { - \sin \upvarphi \cos \uptheta } & {\sin \uptheta } \\ {\sin \upvarphi \cos \uptheta } & 0 & { - \cos \upvarphi \cos \uptheta } \\ { - \sin \uptheta } & {\cos \upvarphi \cos \uptheta } & 0 \\ \end{array} } \right)^{2} \left( {1 - \cos \uppsi } \right) \\ \end{aligned}$$

6 $${\text{T}}_{3} = \left( {\begin{array}{*{20}c} {\begin{array}{*{20}c} {\cos \uppsi } \\ { + \cos^{2} \upvarphi \cos^{2} \uptheta } \\ { - \cos \uppsi \cos^{2} \upvarphi \cos^{2} \uptheta } \\ \end{array} } & {\left[ {\begin{array}{*{20}c} {\cos \upvarphi \sin \uptheta \cos \uptheta } \\ { - \cos \upvarphi \sin \uptheta \cos \uptheta \cos \uppsi } \\ { - \sin \uppsi \cos \uptheta \sin \uppsi } \\ \end{array} } \right]} & {\begin{array}{*{20}c} {\sin \upvarphi \cos \upvarphi \cos^{2} \uptheta } \\ { - \sin \upvarphi \cos \upvarphi \cos^{2} \uptheta \cos \uppsi } \\ { + \sin \uptheta \sin \uppsi } \\ \end{array} } \\ {\left[ {\begin{array}{*{20}c} {\cos \upvarphi \sin \uptheta \cos \uptheta } \\ { - \cos \upvarphi \sin \uptheta \cos \uptheta \cos \uppsi } \\ { + \sin \upvarphi \cos \uptheta \sin \uppsi } \\ \end{array} } \right]} & {\begin{array}{*{20}c} {\cos \uppsi } \\ { + \sin^{2} \uptheta } \\ { - \sin^{2} \uptheta \cos \uppsi } \\ \end{array} } & {\left[ {\begin{array}{*{20}c} {\sin \upvarphi \sin \uptheta \cos \uptheta } \\ { - \sin \upvarphi \sin \uptheta \cos \uptheta \cos \uppsi } \\ { - \cos \upvarphi \cos \uptheta \sin \uppsi } \\ \end{array} } \right]} \\ {\begin{array}{*{20}c} {\sin \upvarphi \cos \upvarphi \cos^{2} \uptheta } \\ { - \sin \upvarphi \cos \upvarphi \cos^{2} \uptheta \cos \uppsi } \\ { - \sin \uptheta \sin \uppsi } \\ \end{array} } & {\left[ {\begin{array}{*{20}c} {\sin \upvarphi \sin \uptheta \cos \uptheta } \\ { - \sin \upvarphi \sin \uptheta \cos \uptheta \cos \uppsi } \\ { + \cos \upvarphi \cos \upvarphi \sin \uppsi } \\ \end{array} } \right]} & {\begin{array}{*{20}c} {\cos \uppsi } \\ { + \sin^{2} \upvarphi \cos^{2} \uptheta } \\ { - \sin^{2} \upvarphi \cos^{2} \uptheta \cos \uppsi } \\ \end{array} } \\ \end{array} } \right)$$

When the three rotational matrices were combined in the following order T1 ⊗ T2 ⊗ T3, the matrix T_4_ simplifies to become:

7 $${\text{T}}_{4} = {\text{T}}_{1} {\text{T}}_{2} {\text{T}}_{3} = \left( {\begin{array}{*{20}c} {\cos \upvarphi \cos \uptheta } & {\sin \uptheta } & {\sin \upvarphi \cos \uptheta } \\ {\sin \upvarphi \sin \uppsi - \cos \upvarphi \sin \uptheta \cos \uppsi } & {\cos \uptheta \cos \uppsi } & { - \cos \upvarphi \sin \uppsi - \sin \upvarphi \sin \uptheta \cos \uppsi } \\ { - \sin \upvarphi \cos \uppsi - \cos \upvarphi \sin \uptheta \sin \uppsi } & {\cos \uptheta \sin \uppsi } & {\cos \upvarphi \cos \uppsi - \sin \upvarphi \sin \uptheta \sin \uppsi } \\ \end{array} } \right)$$

When the transformation is expressed in the form of vectors, *v*_1_ = T_4_ ⋅ *v*_0_

8 $$\left( {\begin{array}{*{20}c} {{\text{x}}_{1} } \\ {{\text{y}}_{1} } \\ {{\text{z}}_{1} } \\ \end{array} } \right) = \left( {\begin{array}{*{20}c} {\cos \upvarphi \cos \uptheta } & {\sin \uptheta } & {\sin \upvarphi \cos \uptheta } \\ {\sin \upvarphi \sin \uppsi - \cos \upvarphi \sin \uptheta \cos \uppsi } & {\cos \uptheta \cos \uppsi } & { - \cos \upvarphi \sin \uppsi - \sin \upvarphi \sin \uptheta \cos \uppsi } \\ { - \sin \upvarphi \cos \uppsi - \cos \upvarphi \sin \uptheta \sin \uppsi } & {\cos \uptheta \sin \uppsi } & {\cos \upvarphi \cos \uppsi - \sin \upvarphi \sin \uptheta \sin \uppsi } \\ \end{array} } \right)\left( {\begin{array}{*{20}c} {{\text{x}}_{0} } \\ {{\text{y}}_{0} } \\ {{\text{z}}_{0} } \\ \end{array} } \right)$$

the vector *v*_1_ can be written in the following explicit form:

9 $$\begin{aligned} {\text{x}}_{1} & = \cos \upvarphi \cos \uptheta {\text{x}}_{0} + \left( {\sin \upvarphi \sin \uppsi - \cos \upvarphi \sin \uptheta \cos \uppsi } \right){\text{y}}_{0} \\ & \quad + \left( { - \sin \upvarphi \cos \uppsi - \cos \upvarphi \sin \uptheta \sin \uppsi } \right){\text{z}}_{0} \\ \end{aligned}$$

10 $${\text{y}}_{1} = \sin \uptheta {\text{x}}_{0} + \cos \uptheta \cos \uppsi {\text{y}}_{0} + \cos \uptheta \sin \uppsi {\text{z}}_{0}$$

11 $$\begin{aligned} {\text{z}}_{1} & = \sin \upvarphi \cos \uptheta {\text{x}}_{0} + \left( { - \cos \upvarphi \sin \uppsi - \sin \upvarphi \sin \uptheta \cos \uppsi } \right){\text{y}}_{0} \\ & \quad + \left( {\cos \upvarphi \cos \uppsi - \sin \upvarphi \sin \uptheta \sin \uppsi } \right){\text{z}}_{0} \\ \end{aligned}$$

Equations 9, 10 and 11 can be used to describe any vector in the space (like how a combination of arbitrary x_0_, y_0_, and z_0_ vectors).

To resolve the three-dimensional rotation of a cylindrical object two vectors are required. So lengthwise and crosswise vectors are introduced to simplifies the equation system. As depicted in Fig. 1, the lengthwise vector, $\overrightarrow{\mathrm{OA}}$, goes from the point located at the center of the implant shoulder, that is point $\mathrm{O}=\left(\mathrm{0,0},0\right)$, to the point in the center of the implant apex, that is the point $\mathrm{A}=\left({\mathrm{A}}_{\mathrm{X}},{\mathrm{A}}_{\mathrm{Y}},{\mathrm{A}}_{\mathrm{Z}}\right)$.

The crosswise vector, $\overrightarrow{\mathrm{OR}}$, goes from the point $\mathrm{O}=\left(\mathrm{0,0},0\right)$ to point $\mathrm{R}=\left({\mathrm{R}}_{\mathrm{X}},{\mathrm{R}}_{\mathrm{Y}},{\mathrm{R}}_{\mathrm{Z}}\right)$.

The method allows clinicians to calculate the implant pose with a known reference object. In this perspective, the center of another implant shoulder (point R of implant 2 in Fig. 1), which has a different pose from the currently analyzed implant (implant 1 in Fig. 1), can be used to disclose the pose rotation along the long axis of the implant 1. Any other fixed reference point can be used in place of the shoulder of implant 2, however (referring object of Fig. 1).

Since the depth in a single two-dimensional image is not taken into account, it's been arbitrarily established that the dimension along X-axis is disregarded. So the system of equations obtained with the known magnitude of $\overrightarrow{\mathrm{OA}}$ and $\overrightarrow{\mathrm{OR}}$ are:

12 $$\overrightarrow {{{\text{OA}}}}_{{\text{Y}}} = \sin {\uptheta A}_{{\text{x}}} + \cos {\uptheta }\cos \uppsi {\text{A}}_{{\text{y}}} + \cos {\uptheta }\sin \uppsi {\text{A}}_{{\text{z}}}$$

13 $$\begin{aligned} \overrightarrow {{{\text{OA}}}}_{{\text{Y}}} & = \sin \upvarphi \cos \uptheta {\text{A}}_{{\text{x}}} + \left( { - \cos \upvarphi \sin \uppsi - \sin \upvarphi \sin \uptheta \cos \uppsi } \right){\text{A}}_{{\text{y}}} \\ & \quad + \left( {\cos \upvarphi \cos \uppsi - \sin \upvarphi \sin \uptheta \sin \uppsi } \right){\text{A}}_{{\text{z}}} \\ \end{aligned}$$

14 $$\overrightarrow {{{\text{OR}}}}_{{\text{Y}}} = \sin \uptheta {\text{R}}_{{\text{x}}} + \cos \uptheta \cos \uppsi {\text{R}}_{{\text{y}}} + \cos \uptheta \sin \uppsi {\text{R}}_{{\text{z}}}$$

15 $$\begin{aligned} \overrightarrow {{{\text{OR}}}}_{{\text{Y}}} & = \sin \upvarphi \cos \uptheta {\text{R}}_{{\text{x}}} + \left( { - \cos \upvarphi \sin \uppsi - \sin \upvarphi \sin \uptheta \cos \uppsi } \right){\text{R}}_{{\text{y}}} \\ & \quad + \left( {\cos \upvarphi \cos \uppsi - \sin \upvarphi \sin \uptheta \sin \uppsi } \right){\text{R}}_{{\text{z}}} \\ \end{aligned}$$

The components viewed from the detector plane and measured within a single two-dimensional radiograph are ${\overrightarrow{\mathrm{OA}}}_{\mathrm{Y}}$,${\overrightarrow{\mathrm{OA}}}_{\mathrm{Z}}$ and ${\overrightarrow{\mathrm{OB}}}_{\mathrm{Y}}$,${\overrightarrow{\mathrm{OB}}}_{\mathrm{Z}}$, and depend on the triad of angles (φ, θ, ψ) and on the vectors’ components $${\overrightarrow{\mathrm{OA}}}_{0}=\left(\begin{array}{c}0\\ 0\\ l\end{array}\right)$$ and $${\overrightarrow{\mathrm{OR}}}_{0}=\left(\begin{array}{c}0\\ {\mathrm{R}}_{\mathrm{Y}}\\ {\mathrm{R}}_{\mathrm{Z}}\end{array}\right)$$The four vector-component equations undergo simplification using the known magnitude of the lengthwise ($\overrightarrow{\mathrm{OA}}$) and crosswise vector ($\overrightarrow{\mathrm{OR}}$).

16 $$\overrightarrow {{{\text{OA}}}}_{{\text{Y}}} = \cos \uptheta \sin \uppsi l$$

17 $$\overrightarrow {{{\text{OA}}}}_{{\text{Z}}} = \left( {\cos \upvarphi \cos \uppsi - \sin \upvarphi \sin \uptheta \sin \uppsi } \right)l$$

18 $$\overrightarrow {{{\text{OR}}}} _{{\text{Y}}} = \cos \uptheta \cos \uppsi {\text{R}}_{{\text{Y}}} + \cos \uptheta \sin \uppsi {\text{R}}_{{\text{Z}}}$$

19 $$\begin{aligned} \overrightarrow {{{\text{OR}}}}_{{\text{Z}}} & = \left( { - \cos \upvarphi \sin \uppsi - \sin \upvarphi \sin \uptheta \cos \uppsi } \right){\text{R}}_{{\text{Y}}} \\ & \quad + \left( {\cos \upvarphi \cos \uppsi - \sin \upvarphi \sin \uptheta \sin \uppsi } \right){\text{R}}_{{\text{Z}}} \\ \end{aligned}$$

in which R_Y_ and R_Z_ can be directly measured by a three-dimensional viewing software (dentascan or CAD/CAM Computer-Aided Design/Computer-Aided Manufacturing technology) or explicitly calculated by Eqs. 20 and 21 as reported in the “Appendix”.

The R_X_ parameter is 0, whereas R_Y_ and R_Z_ are described as the following:

20 $${\mathrm{R}}_{\mathrm{Y}}=\sqrt{\sum_{m=1}^{3}{\left({o}_{m}+\left(\frac{\sum_{n=1}^{3}\left({r}_{n}-{o}_{n}\right)\left({a}_{n}-{o}_{n}\right)}{\sum_{n=1}^{3}{\left({a}_{n}-{o}_{n}\right)}^{2}}\right)\left({a}_{m}-{o}_{m}\right)-{r}_{m}\right)}^{2}}$$

21 $${\mathrm{R}}_{\mathrm{Z}}=\sqrt{\sum_{m=1}^{3}{\left({o}_{m}-{r}_{m}\right)}^{2}-\sum_{m=1}^{3}{\left({o}_{m}+\left(\frac{\sum_{n=1}^{3}\left({r}_{n}-{o}_{n}\right)\left({a}_{n}-{o}_{n}\right)}{{\sum }_{n=1}^{3}{\left({a}_{n}-{o}_{n}\right)}^{2}}\right)\left({a}_{m}-{o}_{m}\right)-{r}_{m}\right)}^{2}}$$

with *n* from 1 to 3, and *m* from 1 to 3 in the interest of brevity of the equations which depend on the three dimensional coordinates of $\mathrm{O}=\left({o}_{1},{o}_{2},{o}_{3}\right)$, $\mathrm{A}=\left({a}_{1},{a}_{2},{a}_{3}\right)$ and $\mathrm{R}=\left({r}_{1},{r}_{2},{r}_{3}\right)$ obtained from either CT or optical scans.

If the marginal bone level (MBL) has to be measured along the $\overrightarrow{\mathrm{OA}}$, a correction factor by using the dental implant itself as internal standard must be employed. The correction factor can be obtained by considering the length of the implant (*len*) expressed by the equation of the magnitude of $\overrightarrow{\mathrm{OA}}$ along the plane of the detector (YZ-plane).

22 $$\overline{{{\text{OA}}_{{{\text{YZ}}}} }} = len \cdot \sqrt {\left( {\cos \uptheta \sin \uppsi } \right)^{2} + \left( {\cos \upvarphi \cos \uppsi - \sin \upvarphi \sin \uptheta \sin \uppsi } \right)^{2} }$$

The value of the correction factor CF is given by Eq. 23:

23 $${\text{MBL}}^{ \wedge } = {\text{MBL}} \cdot \left( {1/\sqrt {\left( {\cos \uptheta \sin \uppsi } \right)^{2} + \left( {\cos \upvarphi \cos \uppsi - \sin \upvarphi \sin \uptheta \sin \uppsi } \right)^{2} } } \right)$$

where MBL and MBL^ are, respectively, the measured and corrected values of the marginal bone level.

The calculated correction factor is identical to that calculated in a previous study [13].

### Experimental evaluation

CT scans and radiographs of patients who had undergone dental implant placement were retrospectively collected from the case sheets between the years 2014 and 2016. Patients were informed about the type of the present in silico study and signed an informed consent form before data selection and analysis and approval for this retrospective analysis was obtained from the local Ethical Committee. Implants were usually placed according to the instructions of the manufacturers employing an electric handpiece, electro-magnetic (Magnetic Mallet, Osseotouch www.osseotouch.com, Turbigo, Milano, Italy) and piezo-electric devices (Piezosurgery, Mectron, Carasco, Italy). Ethics approval was not required for this in vitro study. The present paper was prepared according to the SQUIRE2.0 checklist.

The cone-beam computerized tomography scan (Gendex GXCB-500, Gendex Dental Systems) provides a finely-detailed three-dimensional overview of the bony architecture around the implants inserted in it. The following set of default parameters have been applied in the acquisition of scans: 120 kV, 30.89 mAs, 0.2 mm × 0.2 mm × 0.2 mm isotropic voxel size, and 8.72 mm diameter of Field Of View. Digital intra-oral periapical radiographs were taken (70 kVp, 7 mA) using a CMOS (Complementary Metal Oxide Semiconductor) digital sensor (Schick CDR Elite, Schick Technologies Inc., Long Island City, NY, USA) and imaging software (FONA Computed Dental Radiography DICOM 4.5, Schick Technologies Inc., Long Island City, NY, USA) with a pixel pitch of 100 μm (0.1 mm × 0.1 mm; bit depth, 8-bit grayscale).

### Experimental simulation

Experimental evidence that supported the validity of the abovementioned vector equations and verified the model's accuracy was given by an algorithm using components ${\overrightarrow{\mathrm{OA}}}_{\mathrm{Y}}$, ${\overrightarrow{\mathrm{OA}}}_{\mathrm{Z}}$, ${\overrightarrow{\mathrm{OR}}}_{\mathrm{Y}}$, ${\overrightarrow{\mathrm{OR}}}_{\mathrm{Z}}$ directly measured on the radiographs as per in Fig. 1 and “Appendix”. Digital prototypes of cylinder screwed, root-form, 1 mm-tread pitch dental implants were obtained by a Cone Beam Computerized Tomography scanner (Gendex GXCB-500, Gendex Dental Systems) with the following setting: 120 kV, 30.89 mAs, isotropic voxel size of 200 μm. The virtual 3D phantom-implant was voxelized and interpolated from the original.dcm file with the following setting: 100 μm × 100 μm × 100 μm, bit depth 8-bit grayscale.

A simulated radiograph could be generated through the overlapping of all the phantom-layers depicting implant in three-dimensions along the direction of the x-axis, so obtaining a projected phantom-implant in the YZ-plane (like it is the detector plane) by a subroutine described in a preceding article [3].

### Variables

#### Determination of numerical variables

For each dental implant, a dentascan program was used to acquire the three-dimensional coordinates of the three reference points A, O, and R to calculate *l*, R_Y_, and R_Z_, that is the magnitude of the vectors (${\overrightarrow{\mathrm{OA}}}_{0}$ and ${\overrightarrow{\mathrm{OR}}}_{0}$), in other words, input parameters to be inserted in the right-hand side of equations from 16 to 19. A free standalone software (Osiris 4.19 the University of Genève. Switzerland) applying a mouse-driven measurement tool was used to acquire the two-dimensional coordinates of the points A, O, and R to calculate the components ${\overrightarrow{\mathrm{OA}}}_{\mathrm{Y}}$,${\overrightarrow{\mathrm{OA}}}_{\mathrm{Z}}$ and ${\overrightarrow{\mathrm{OR}}}_{\mathrm{Y}}$,${\overrightarrow{\mathrm{OR}}}_{\mathrm{Z}}$, that is, input parameters to be inserted in the left-hand side of equations from 16 to 19. Measurements of coordinates and components were taken twice by two independent investigators (CS, PDM). The diameter of the dental implant at healing cap/implant-abutment connection (∅ from 4 to 5 mm), which was one of the cleanest and more consistent features to be certainly identified in a cylindrical shaped object, allowed the investigators to calibrate all the images. Numbers in vectors analysis provided a resolution of a hundredth of a millimeter.

#### Pose angle triplet

One set of angles (triad) was given by a triplet of rotational angles φ, θ and ψ (pitch, roll, and yaw, respectively). The position of each radiographic cylindrical dental implant could be described by a radiographic triad of angles (_*r*_φ, _*r*_θ, _*r*_ψ) which was estimated by numerically solving the system of equations from 16 to 19 by using the algorithm suggested by Toti et al. [13], radiographic components ${{}_{r}\overrightarrow{\mathrm{OA}}}_{\mathrm{Y}}$, ${}_{r}{\overrightarrow{\mathrm{OA}}}_{\mathrm{Z}}$, ${}_{r}{\overrightarrow{\mathrm{OR}}}_{\mathrm{Y}}$, ${}_{r}{\overrightarrow{\mathrm{OR}}}_{\mathrm{Z}}$, and following setting for the algorithms running on a matrix elaborator (MatLab 7.13, The MathWorks, Natick, MA, USA): “Levenberg–Marquardt” ON.

**Fig. 2:**
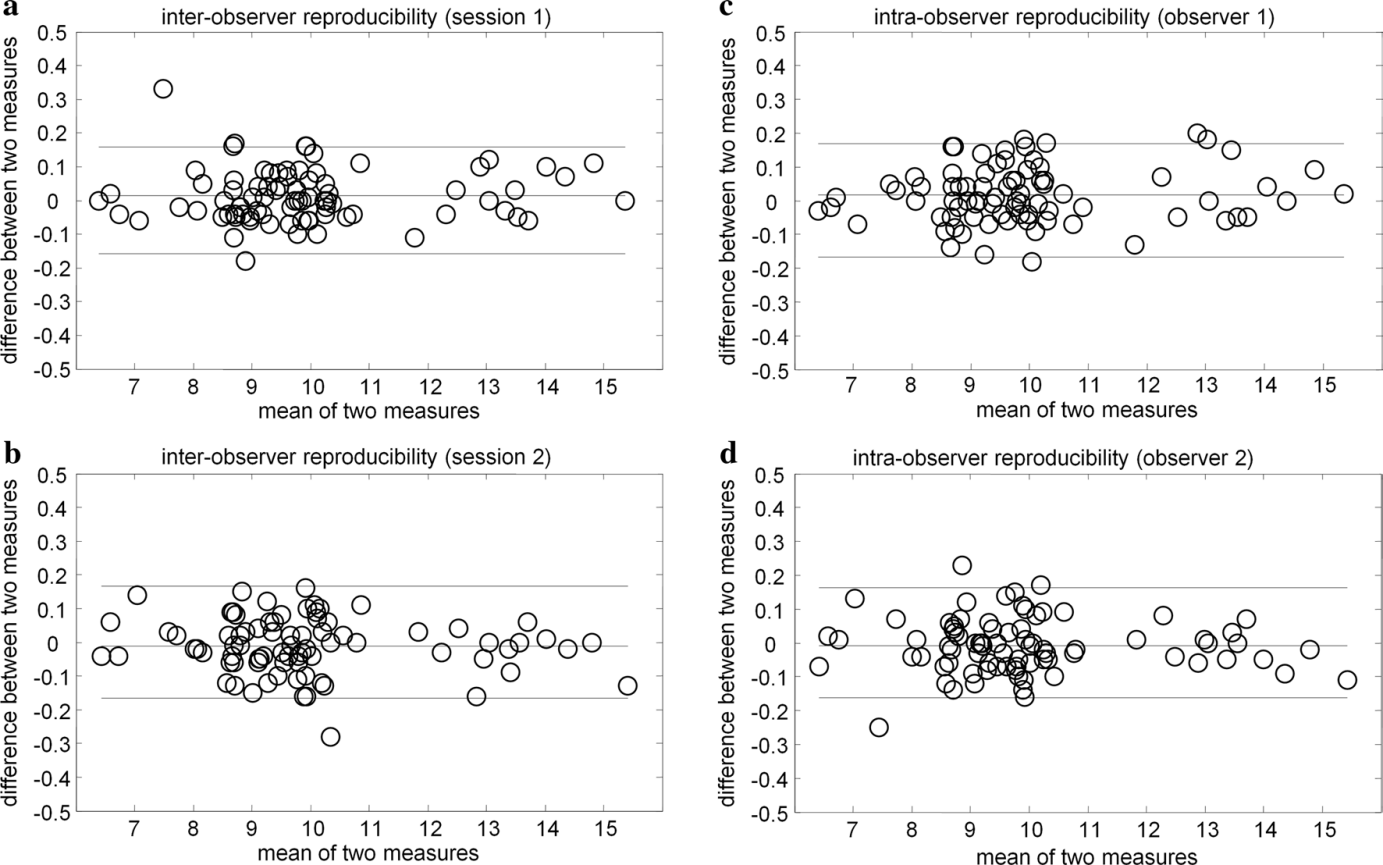
**a**, **b** Inter-onserver and **c**, **d** Intra-observer reproducibility (Bland–Altman plots) of the measured lengths in millimeters of the two vectors $\overrightarrow{\mathrm{OA}}$ and $\overrightarrow{\mathrm{OR}}$. The straight horizontal line represents the mean difference between sessions and the dashed horizontal lines represent the limits of agreement (95% confidence interval)

For each radiographic implant (with input: length, diameter triad of angles, and referring points) a projected phantom of a dental implant was generated; analyzing simulated images, simulated components ${{}_{s}\overrightarrow{\mathrm{OA}}}_{\mathrm{Y}}$,${}_{s}{\overrightarrow{\mathrm{OA}}}_{\mathrm{Z}}$,${}_{s}{\overrightarrow{\mathrm{OR}}}_{\mathrm{Y}}$,${}_{s}{\overrightarrow{\mathrm{OR}}}_{\mathrm{Z}}$ were obtained, and the simulated triad of angles (_*s*_φ, _*s*_θ, _*s*_ψ) was calculated as shown in Fig. 2. So radiographic and simulated triads could be statistically compared.

### Statistical analysis

The solution of the system of equations was computed with a specific algorithm (MatLab7.13, The MathWorks, Natick, MA, USA). The correlation between the radiographic length of implants and that simulated by using the angular correction factor of implants was tested by robust linear regression. Inter- and intra-observer agreements were tested by Bland–Altman analysis. Absolute differences between the radiographic and virtual triad of angles (test of accuracy) were calculated.

## Results

Twenty intraoral radiographs (10 in the upper- and 10 in the lower-jaw) were selected and analyzed for the present in silico retrospective study. Y- and Z-components of the two-directional vectors $\overrightarrow{\mathrm{OA}}$ and $\overrightarrow{\mathrm{OR}}$ were measured for each selected cylindrical dental implant. The components were used in a non-linear fitting procedure, and triads of angles—describing the rotations—were estimated. Table 1 shows the values of the components of the estimated angles with the results of statistical analysis.

**Table 1:** Measured- (*radiographic*) and simulated-values (*simulated*) of the components of vector $\overrightarrow{\mathrm{OA}}$ and vector $\overrightarrow{\mathrm{OR}}$ with related values of rotational angles

Radiograph		Components (mm)				Angles (°)			Optimization metric						
		${\overrightarrow{OA}}_{Y}$	${\overrightarrow{OA}}_{Z}$	${\overrightarrow{OR}}_{Y}$	${\overrightarrow{OR}}_{Z}$	φ	θ	ψ	Iterations		Residual		First-order optimality		Status of equation
		Mean	Mean	Mean	Mean	Mean	Mean	Mean	Min	Max	Min	Max	Min	Max	
1	Radiographic	0.04	9.06	12.57	− 2.75	− 15.90	31.98	1.45	6	7	0.0548	0.4935	− 2.5⋅10^−10^	− 8.6⋅10^−13^	Inaccuracy
	Simulated	− 0.13	9.11	12.28	− 2.36	− 13.65	34.30	2.43	5	7	0.4865	0.5778	− 4.8⋅10^−11^	− 1.4⋅10^−12^	Inaccuracy
2	Radiographic	− 0.26	9.30	12.86	1.92	20.48	30.48	1.68	4	5	2⋅10^−05^	0.0008	− 9.0⋅10^−12^	− 1.4⋅10^−13^	Solved
	Simulated	− 0.21	8.82	12.80	2.56	26.73	30.68	1.08	5	5	0.0029	0.0214	− 4.7⋅10^−11^	− 1.2⋅10^−13^	Inaccuracy
3	Radiographic	3.45	9.33	12.10	− 5.64	10.80	24.58	− 22.43	6	7	3⋅10^−05^	0.0109	− 2.7⋅10^−11^	− 1.0⋅10^−16^	Inaccuracy
	Simulated	3.52	9.21	12.40	− 5.21	16.05	21.23	− 22.45	6	7	0.0014	0.0082	− 1.1⋅10^−11^	− 1.1⋅10^−13^	Solved
4	Radiographic	− 3.61	9.20	9.20	1.61	− 27.63	46.23	31.85	5	5	0.0005	0.0185	− 5.9⋅10^−11^	− 1.4⋅10^−12^	Inaccuracy
	Simulated	− 3.73	9.54	9.47	2.21	− 24.05	45.00	30.98	5	6	0.0280	0.0880	− 5.1⋅10^−11^	− 5.9⋅10^−14^	Inaccuracy
5	Radiographic	− 3.63	8.43	4.67	5.91	− 26.33	62.40	52.30	6	6	0.0050	0.2729	− 3.7⋅10^−11^	− 1.4⋅10^−13^	Inaccuracy
	Simulated	− 3.77	8.40	4.55	6.67	− 24.80	62.23	53.80	6	6	0.0001	0.0049	− 7.2⋅10^−14^	− 9.2⋅10^−17^	Solved
6	Radiographic	4.58	9.20	9.33	− 5.54	24.40	37.15	− 33.88	7	7	0.0501	0.1126	− 5.1⋅10^−11^	− 3.8⋅10^−12^	Inaccuracy
	Simulated	4.27	8.04	9.13	− 4.83	31.73	36.05	− 35.93	9	9	0.6050	0.7298	− 1.9⋅10^−11^	− 6.1⋅10^−12^	Inaccuracy
7	Radiographic	− 2.01	6.28	10.66	− 5.10	− 57.45	39.33	19.85	6	7	1.0931	1.3395	− 2.7⋅10^−10^	− 5.6⋅10^−12^	Unsolved
	Simulated	− 2.57	6.57	11.08	− 5.28	− 58.50	38.43	20.78	5	5	0.1128	0.1750	− 2.0⋅10^−10^	− 5.9⋅10^−11^	Inaccuracy
8	Radiographic	2.77	8.35	14.04	− 3.03	33.05	9.00	− 15.35	8	8	0.0296	0.1085	− 2.6⋅10^−11^	− 1.5⋅10^−13^	Inaccuracy
	Simulated	2.51	7.76	13.76	− 2.69	38.60	8.73	− 15.35	7	7	0.0142	0.0627	− 1.6⋅10^−10^	− 9.5⋅10^−13^	Inaccuracy
9	Radiographic	− 1.95	9.40	13.62	1.52	− 6.18	22.93	12.88	5	10	0.1002	0.2357	− 9.5⋅10^−11^	− 2.4⋅10^−12^	Inaccuracy
	Simulated	− 2.23	9.38	13.41	1.91	− 6.70	24.30	14.73	6	8	0.1079	0.1540	− 8.8⋅10^−11^	− 3.7⋅10^−12^	Inaccuracy
10	Radiographic	4.63	7.30	13.03	− 7.06	− 32.88	4.45	− 26.50	9	10	0.0451	0.1590	− 1.5⋅10^−01^	− 2.3⋅10^−13^	Inaccuracy
	Simulated	4.59	7.37	13.78	− 6.85	− 32.90	4.95	− 24.58	9	12	0.7022	0.9830	− 7.8⋅10^−11^	− 4.7⋅10^−12^	Inaccuracy
11	Radiographic	− 5.05	− 8.36	− 9.45	3.96	9.88	− 10.38	148.23	15	17	0.1892	0.2592	− 1.2⋅10^−11^	− 7.8⋅10^−12^	Inaccuracy
	Simulated	− 5.60	− 8.53	− 9.98	4.00	0.00	0.00	147.13	16	42	0.7688	0.8621	− 0.2570	1.2⋅10^−08^	Inaccuracy
12	Radiographic	− 1.87	− 8.66	− 10.43	1.80	26.53	− 14.15	166.73	12	14	1.0170	1.1464	− 2.9⋅10^−11^	− 5.1⋅10^−12^	Unsolved
	Simulated	− 2.17	− 8.67	− 9.86	1.22	24.85	− 12.13	166.78	8	9	0.0224	0.0686	− 4.6⋅10^−12^	− 7.5⋅10^−13^	Inaccuracy
13	Radiographic	− 0.16	− 9.93	− 8.66	− 0.96	− 13.13	27.08	180.55	9	10	0.0213	0.0963	− 4.1⋅10^−11^	− 4.2⋅10^−13^	Inaccuracy
	Simulated	0.13	− 9.62	− 8.66	− 1.05	− 12.00	26.63	179.58	9	10	0.0252	0.0549	− 1.9⋅10^−11^	− 2.5⋅10^−12^	Inaccuracy
14	Radiographic	0.51	− 8.64	− 6.16	1.81	− 34.68	49.80	184.43	7	7	0.0002	0.0098	− 1.1⋅10^−12^	− 3.9⋅10^−15^	Solved
	Simulated	0.39	− 8.53	− 6.47	1.87	− 34.05	47.53	183.20	6	7	8⋅10^−06^	0.0163	− 6.5⋅10^−11^	− 4.1⋅10^−14^	Inaccuracy
15	Radiographic	− 3.31	− 9.67	− 8.69	0.14	17.08	27.63	159.08	8	9	0.0296	0.1491	− 4.6⋅10^−11^	− 9.4⋅10^−13^	Inaccuracy
	Simulated	− 3.33	− 9.25	− 9.26	0.35	19.33	19.20	159.55	6	8	4⋅10^−05^	0.0222	− 1.8⋅10^−11^	− 2.9⋅10^−12^	Inaccuracy
16	Radiographic	− 3.74	− 9.16	− 7.08	3.11	1.40	41.20	150.30	8	9	0.1664	0.2373	− 9.7⋅10^−11^	− 1.6⋅10^−12^	Inaccuracy
	Simulated	− 3.77	− 8.60	− 7.38	3.16	− 2.35	38.99	151.10	7	8	1⋅10^−06^	0.0083	− 1.7⋅10^−12^	− 7.9⋅10^−15^	Solved
17	Radiographic	− 3.44	− 9.42	− 9.75	0.79	− 13.88	− 10.80	160.45	8	12	0.0374	0.0944	− 5.0⋅10^−12^	− 7.3⋅10^−13^	Inaccuracy
	Simulated	− 3.42	− 9.44	− 10.07	0.92	− 10.93	− 5.08	161.15	13	21	0.1083	0.1725	− 2.2⋅10^−11^	− 2.2⋅10^−12^	Inaccuracy
18	Radiographic	1.44	− 9.85	− 10.23	− 1.59	18.78	− 10.00	184.40	16	18	1.4629	1.6826	− 8.4⋅10^−12^	− 7.4⋅10^−13^	Unsolved
	Simulated	0.96	− 9.44	− 9.87	− 2.12	20.25	− 5.60	184.30	12	14	0.1740	0.2635	− 2.9⋅10^−11^	− 4.2⋅10^−12^	Inaccuracy
19	Radiographic	− 1.98	− 8.48	− 7.86	3.39	23.10	− 38.70	164.18	8	8	0.2027	0.2352	− 6.7⋅10^−13^	− 9.2⋅10^−14^	Inaccuracy
	Simulated	− 2.15	− 8.40	− 8.28	3.51	24.23	− 35.25	163.35	7	8	0.2834	0.3826	− 3.7⋅10^−11^	− 2.4⋅10^−12^	Inaccuracy
20	Radiographic	2.77	− 9.16	− 9.04	− 1.84	30.15	− 18.55	193.43	10	12	0.7990	0.9605	− 9.6⋅10^−11^	− 1.5⋅10^−12^	Inaccuracy
	Simulated	2.58	− 9.09	− 9.48	− 2.40	27.65	− 11.80	192.18	11	11	0.7052	0.8434	− 3.0⋅10^−11^	− 3.4⋅10^−12^	Inaccuracy

Results of the fitting (status of the fitting) with the optimization metric, that is, the output of the variables those the machine by using “Levenberg–Marquardt algorithm” tried to minimize so that you end up with the best performing machine models: system of equations *solved* if residuals ≤ 10^–2^; possible inaccuracy in solution if 10^–2^ < residuals ≤ 1; system of equations *unsolved* if residuals > 1

Among all the fitting results (40), 37 resulted as resolved with residuals ≤ 1 mm, whereas 32 of them reported on the possible inaccuracies. Results for the 3 non-linear fittings indicated unsolved equations with residuals > 1 mm. However, the system was well over determined (more equations, four, than unknowns, three).

**Fig. 3:**
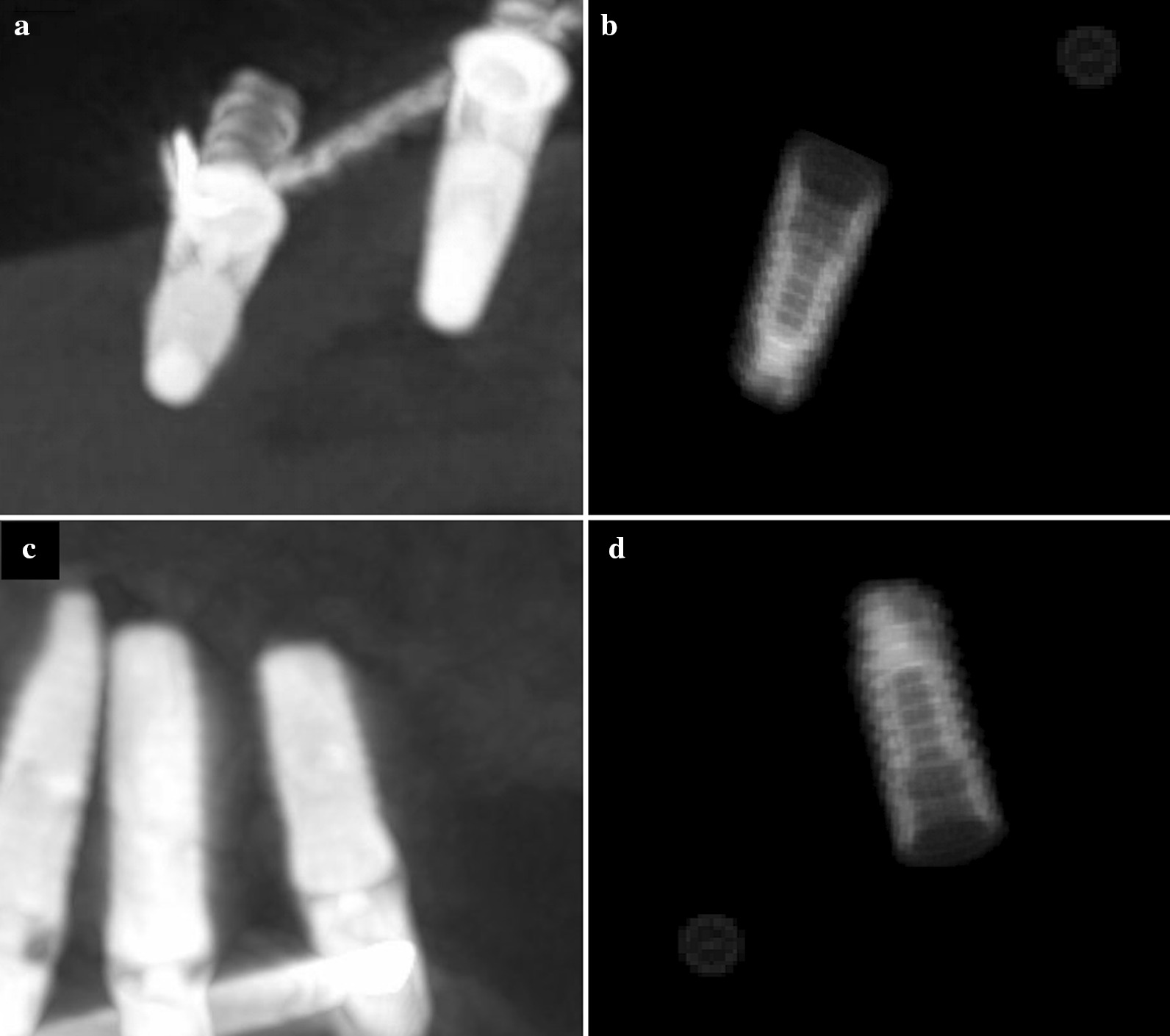
Radiographs of lower and upper arch dental implants (on the right: **a**, **c**) and related virtual phantom with respective referring points (on the left: **b**, **d**)

As was to be expected, how angles and rotations had been set out in the theoretical section indicated that in the upper-jaw implants ψ might be of values close to 180° (downwards, in the range between 148.2° and 193.4° with a mean of 169.2°), whereas in the lower-jaw implants ψ ranged from − 33.9° to 52.3° with a mean of 2.2 (upwards) (Fig. 3).

### Reproducibility of the angular measurements

Intra-examiner and inter-examiner reproducibility were described in detail with Bland and Altman plots in Fig. 2. The intra-examiner differences ranged from − 0.18 to + 0.2 mm for observer 1, and from − 0.25 to + 0.25 mm for observer 2, respectively. The inter-examiner differences ranged from − 0.18 to + 0.33 mm for session 1, and from − 0.28 to + 0.16 mm for session 2, respectively.

### Accuracy of the angular measurements

**Fig. 4:**
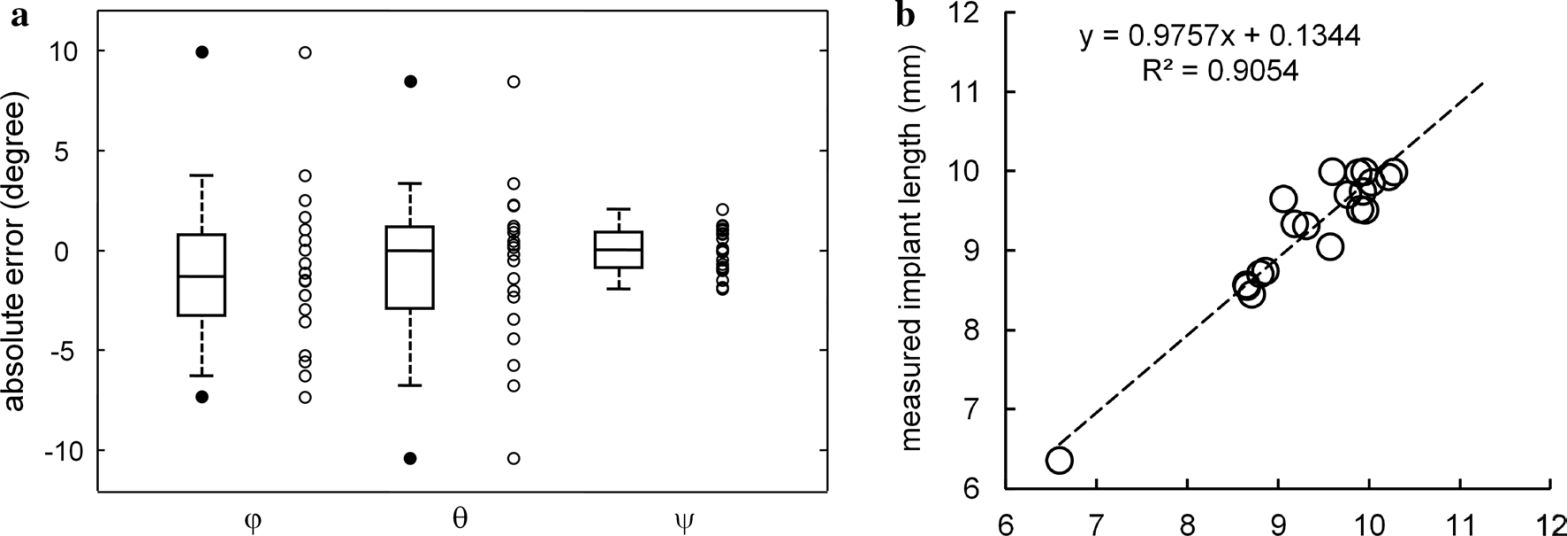
**a** Accuracy was measured as the difference between two sets of angles (three angles of rotations) obtained comparing cylindrical implants on real radiographs versus phantom projection on virtual radiographs. Data were represented as scatter (empty points) and box-and-whisker plot (the box line represents the lower. median. and upper quartile values; the whisker lines include the rest of the data). Outliers (solid points) were data with values beyond the ends of the whiskers. **b** Linear dependence between registered length of the implant as it appears in the radiograph and length as obtained by means of the angular correction factor (CF). The dashed line represents the robust fit (equation and adjusted coefficient of determination are indicated in the graph)

The description of the output triad of angles for real radiographs was of − 7.8° ± 29.3°, 30.9° ± 17.1°, and 2.2° ± 27.6° respectively for angles φ, θ, and ψ in the lower jaw, whereas was of 6.5° ± 21.2°, 4.3° ± 29.5°, and 169.2° ± 15.6° respectively for angles φ, θ, and ψ in the upper jaw. Very similar values were obtained for the simulated triad of angles, as they appear in the virtual phantoms: − 4.8° ± 31.9° and 5.7° ± 20.7° for angle φ; 30.6° ± 16.9° and 6.3° ± 25.9° for angle θ; 2.6° ± 28.0° and 168.8° ± 15.2° for angle ψ, for the lower- and the upper-jaws, respectively. Absolute error, as shown in Fig. 4, was: − 1.1° ± 3.9° for angle φ; − 0.9° ± 4.1° for angle θ; 0° ± 1.1° for angle ψ.

### Correction factors for marginal bone level

Radiographs with the related phantoms of cylindrical dental implants, simulated by using the results of triad-fitting, are shown in Fig. 2. As shown in Fig. 4, radiographic lengths of the implant as measured on the radiographs and simulated lengths calculated with the angular correction factors appeared to be heavily correlated. Such a linear dependence was verified by the results of robust linear regression, which employed an iteratively reweighted least-squares algorithm less sensitive to outliers. All collected data were included without any data exclusion and the following coefficients resulted 0.9757 (slope) and + 0.1344 (intercept), with an adjusted coefficient of determination of 0.9054.

## Discussion

The number of degrees of freedom in intra-oral radiograph describing the projection of an object on the detector’s surface could be reduced by the use of devices for image standardization, that is, bite block with fixed-length extension cone paralleling instruments coupled with equipment indicating the direction of the dental x-ray tube. In this way, the degrees of freedom went from nine to six; moreover, those related to the object’s translation in space had a negligible effect because the dental implant was the reference object for the radiograph.

The basic idea of the present study was that a cylindrical object did not have enough information, at this stage, to resolve all degrees of freedom about the position and direction of the dental implant.

Several authors attempted to reduce the number of degrees of freedom to simplify the analysis of the physical system. Some authors let the coordinate system be located external to the relevant structures using at least three radiopaque spheres of known dimension [4–6]; one of them later had been quite successful in achieving a solution to the descriptions of the geometric structure about the position of the cylindrical dental implant in space [12]; he designed the system of the rigid object to be as compact as possible while describing translational and rotational vector graphics. However, the degree of freedom related to the rotation along the main axis of the dental implant remained unknown. The author suggested that lost rotation might be solved by simply adding an extra reference point not collinear with the dental implant axis. Theoretically, a known pose of the cylindrical dental implant through three reference points, two inside the implant body, and the third in a mesial or distal position (further dental implant, tooth, or identifiable anatomic landmark) reduced all degrees of freedom to one.

In a previous paper, Toti and co-workers suggested that “vector analysis” attempted to estimate the pose of a blade implant [13]. It should be noted that components of the lengthwise vector were the same as those reported in Eqs. 6 and 7; the only adjustment was the value of the parameter *l* which represented the length of the cylindrical dental implant. On the contrary, the y and z components of the crosswise vector had more complex equations in which the two terms (R_Y_ and R_Z_) depended on both the three-dimensional position of the third reference point (R) and the triad of angles (φ, θ, ψ).

The system given by equations from 6 to 9 admitted explicit solutions; thereby disregarding solutions could be doubled or quadrupled if inverse trigonometric functions were introduced. An equation solver allowed clinicians to obtain very easily an explicit solution given in terms of a single triad of angles. The measurements of fixed parameters (*l*, R_Y_, R_Z_) and input variables (${\overrightarrow{\mathrm{OA}}}_{\mathrm{Y}}$,${\overrightarrow{\mathrm{OA}}}_{\mathrm{Z}}$,${\overrightarrow{\mathrm{OR}}}_{\mathrm{Y}}$,${\overrightarrow{\mathrm{OR}}}_{\mathrm{Z}}$) appeared to give reproducible and reliable results when analyses had been performed with Bland and Altman test (inter- and intra-observer differences ranging from − 0.28 mm to + 0.33 mm).

Unfortunately, the present formulation did not take into account the distorting effects produced by the misalignment between the detector ring radius and the x-ray beam caused by neglect of radiological guidelines or involuntary patient movement [14]. Even if a theoretical approach to assess the quality of the image standardization had not yet been developed for a cylindrical object, data points on implant lengths radiographically measured and then calculated using the angular correction factors (Eq. 11) well matched the straight line, so the assumption of the linear dependence was justified and the linear regression model could be applied with acceptable accuracy (value of the adjusted coefficient of determination of 0.9054).

Results of non-linear fittings accounting for differences between radiographic (_*r*_φ, _*r*_θ, _*r*_ψ) and a simulated triad of angles (_*s*_φ, _*s*_θ, _*s*_ψ) showed that absolute errors had means ranging from − 1.1° to 0° with low measures of dispersion (e.g. standard deviations ≤ 4.1°). The results were in agreement with those described in a previous paper (absolute error from − 0.29 to + 0.64 where 4.35° was the maximum standard deviation) [13].

And if anyone asked where experimental errors arose, it’s supposed to be caused by the cylindrical shape of the dental implant for which an exact calculation of the coordinates of three referring points (O, A, and R) could difficulty result. Moreover, virtual phantom obtained from two-dimensional radiographic simulation had not high enough resolution for such application (pixel resolution of 0.05 mm). Another source of error in the calculation of components could arise from the divergence between the x-ray beam and the normal vector to the detector surface. The problem of the misalignment could not yet be resolved a posteriori for a cylindrical system, at least so far.

Cliniciancs need to be especially mindful to understand that all the processes implemented using algorithms [13], such as measurements of the components of the lengthwise and crosswise vectors and angles calculation of the pose or virtual simulation of the pose of the cylindrical implant are completely independent from each other; differences between true and simulated could only be due to measurement errors (and maybe discretization errors due to the pixel values).

Instead of making a radiograph under known geometric conditions, then computing the pose of the implant from it, and then comparing the pose to the known pose in the imaging geometry, the authors decided to directly measure the pose of the implant on a real radiograph and to simulate obtained angular results. This is because it is very difficult to experimentally check the position of a cylindrical object through three angles one of which including the component describing a rotation of the object along the main axis (hidden component). Moreover, there is also a slight misassumption included in the definition of the projection of the real-world points O, A, and R: due to the projection geometry, the center of the elliptical shadow cast by the (upper) end of the cylinder does not coincide with the real-world center-point along the longitudinal axis (A). However, the influence of this misassumption on the accuracy of the method is expected to be negligible, as for common geometries describing the difference could be of sub-pixel magnitude.

Externalization of the present results (system of Eqs. 16–19) could be used for pose determination of any conical/cylindrical object observed in two-dimensional radiographs. For example, the present method to evaluate the angulations of teeth, knowing their length and through the use of only one referring point, offered an interesting perspective in the fields of orthodontics.

There is no doubt that any improvement in the resolution of the detector and automated detection of parameters and components could help refine the method. The authors are trying to solve equations involving parameters related to errors in alignment.

There are limitations in the present study. The main concern is the small sample size with only twenty intraoral radiographs (ten each from upper and lower jaw) that may be not enough to have confirmative conclusions. Another potential bias is that the intraoral radiographs were taken from anterior or posterior regions with possible different results due to different bone thickness.

## Conclusions

Theoretical and experimental methods, estimating the pose of a cylindrical dental implant in three-dimensional space allowed clinicians to calculate a posteriori single triad of angles (φ, θ, ψ) by analyzing two-dimensional images. The value of the correction factor calculated based on data obtained by analysis of angles supplies the clinician with additional information for the real measurement of the implant marginal bone level. The a posteriori standardization of two-dimensional radiographs could allow the clinicians to minimize the patient's exposure to ionizing radiations for the measurement of success or failure rates. Finally, information about the rotation of a cylindrical like structure (tooth) could be useful in various fields of dentistry.

## Data Availability

All data generated and analysed during this study are published in Table 1 and figures, plus all data generated are available from the corresponding author upon reasonable request.

## Appendix

### The crosswire vector

A line thorough $O=\left({o}_{1},{o}_{2},{o}_{3}\right)$ and $A=\left({a}_{1},{a}_{2},{a}_{3}\right)$ is formed by points $P=\left(x,y,z\right)$ for which vector $\overrightarrow{OP}$ has the same direction of the vector $\overrightarrow{AO}$ as per

24 $$\overrightarrow{OP}=t\overrightarrow{AO} (\mathrm{Eq}.\,1,\;{\mathrm{with}}\;t\;{\mathrm{real}})$$

So

25 $$\left(x-{o}_{1},y-{o}_{2},z-{o}_{3}\right)=\left(t\left({a}_{1}-{o}_{1}\right),t\left({a}_{2}-{o}_{2}\right),t\left({a}_{3}-{o}_{3}\right)\right).$$

And the system of coordinates for P is

26 $$\left\{\begin{array}{c}x={o}_{1}+t\left({a}_{1}-{o}_{1}\right)\\ y={o}_{2}+t\left({a}_{2}-{o}_{2}\right)\\ z={o}_{3}+t\left({a}_{3}-{o}_{3}\right)\end{array}\right.$$

The plane perpendicular to the vector $\overrightarrow{AO}$ and passing through $R=\left({r}_{1},{r}_{2},{r}_{3}\right)$ is

27 $$\left({a}_{1}-{o}_{1},{a}_{2}-{o}_{2},{a}_{3}-{o}_{3}\right)\cdot \left(x-{r}_{1},y-{r}_{2},z-{r}_{3}\right)=0$$

from which Cartesian equation of the plane is the following:

28 $$\left({a}_{1}-{o}_{1}\right)x-{r}_{1}\left({a}_{1}-{o}_{1}\right)+\left({a}_{2}-{o}_{2}\right)y-{r}_{2}\left({a}_{2}-{o}_{2}\right)+\left({a}_{3}-{o}_{3}\right)z-{r}_{3}\left({a}_{3}-{o}_{3}\right)=0$$

Then equation of the straight line intersecting the piano in the point H can be obtained by the following system:

29 $$\left\{\begin{array}{c}\begin{array}{l}x={o}_{1}+t\left({a}_{1}-{o}_{1}\right)\\ y={o}_{2}+t\left({a}_{2}-{o}_{2}\right)\\ z={o}_{3}+t\left({a}_{3}-{o}_{3}\right)\end{array} \\ \left({a}_{1}-{o}_{1}\right)x-{r}_{1}\left({a}_{1}-{o}_{1}\right)+\left({a}_{2}-{o}_{2}\right)y-{r}_{2}\left({a}_{2}-{o}_{2}\right)+\left({a}_{3}-{o}_{3}\right)z-{r}_{3}\left({a}_{3}-{o}_{3}\right)=0\end{array}\right.$$

in which t has the following equation:

30 $$t=\frac{{r}_{1}\left({a}_{1}-{o}_{1}\right)-{o}_{1}\left({a}_{1}-{o}_{1}\right)+{r}_{2}\left({a}_{2}-{o}_{2}\right)-{o}_{2}\left({a}_{2}-{o}_{2}\right)+{r}_{3}\left({a}_{3}-{o}_{3}\right)-{o}_{3}\left({a}_{3}-{o}_{3}\right)}{\left({\left({a}_{1}-{o}_{1}\right)}^{2}+{\left({a}_{2}-{o}_{2}\right)}^{2}+{\left({a}_{3}-{o}_{3}\right)}^{2}\right)}$$

and the point $H=\left({h}_{1},{h}_{2},{h}_{3}\right)$ has the following coordinates:

31 $$\left\{\begin{array}{c}{h}_{1}={o}_{1}+\left(\frac{{r}_{1}\left({a}_{1}-{o}_{1}\right)-{o}_{1}\left({a}_{1}-{o}_{1}\right)+{r}_{2}\left({a}_{2}-{o}_{2}\right)-{o}_{2}\left({a}_{2}-{o}_{2}\right)+{r}_{3}\left({a}_{3}-{o}_{3}\right)-{o}_{3}\left({a}_{3}-{o}_{3}\right)}{\left({\left({a}_{1}-{o}_{1}\right)}^{2}+{\left({a}_{2}-{o}_{2}\right)}^{2}+{\left({a}_{3}-{o}_{3}\right)}^{2}\right)}\right)\left({a}_{1}-{o}_{1}\right)\\ {h}_{2}={o}_{2}+\left(\frac{{r}_{1}\left({a}_{1}-{o}_{1}\right)-{o}_{1}\left({a}_{1}-{o}_{1}\right)+{r}_{2}\left({a}_{2}-{o}_{2}\right)-{o}_{2}\left({a}_{2}-{o}_{2}\right)+{r}_{3}\left({a}_{3}-{o}_{3}\right)-{o}_{3}\left({a}_{3}-{o}_{3}\right)}{\left({\left({a}_{1}-{o}_{1}\right)}^{2}+{\left({a}_{2}-{o}_{2}\right)}^{2}+{\left({a}_{3}-{o}_{3}\right)}^{2}\right)}\right)\left({a}_{2}-{o}_{2}\right)\\ {h}_{3}={o}_{3}+\left(\frac{{r}_{1}\left({a}_{1}-{o}_{1}\right)-{o}_{1}\left({a}_{1}-{o}_{1}\right)+{r}_{2}\left({a}_{2}-{o}_{2}\right)-{o}_{2}\left({a}_{2}-{o}_{2}\right)+{r}_{3}\left({a}_{3}-{o}_{3}\right)-{o}_{3}\left({a}_{3}-{o}_{3}\right)}{\left({\left({a}_{1}-{o}_{1}\right)}^{2}+{\left({a}_{2}-{o}_{2}\right)}^{2}+{\left({a}_{3}-{o}_{3}\right)}^{2}\right)}\right)\left({a}_{3}-{o}_{3}\right)\end{array}\right.$$

The Y coordinate is the distance between H and R that is:

32 $$Y=\sqrt{{\left({h}_{1}-{r}_{1}\right)}^{2}+{\left({h}_{2}-{r}_{2}\right)}^{2}+{\left({h}_{3}-{r}_{3}\right)}^{2}}$$

33 $$Y=\sqrt{\sum_{n=1}^{3}{\left({h}_{n}-{r}_{n}\right)}^{2}}$$

And the coordinate Z is:

34 $$Z=\sqrt{\left({\left({o}_{1}-{r}_{1}\right)}^{2}+{\left({o}_{2}-{r}_{2}\right)}^{2}+{\left({o}_{3}-{r}_{3}\right)}^{2}\right)-\left({\left({h}_{1}-{r}_{1}\right)}^{2}+{\left({h}_{2}-{r}_{2}\right)}^{2}+{\left({h}_{3}-{r}_{3}\right)}^{2}\right)}$$

or

35 $$Z=\sqrt{\sum_{n=1}^{3}{\left({o}_{n}-{r}_{n}\right)}^{2}-\sum_{n=1}^{3}{\left({h}_{n}-{r}_{n}\right)}^{2}}$$

and explicit magnitude equations of the initial crosswise vector ${\overrightarrow{OR}}_{0}$ from the point O (0, 0, 0) to point R (R_X_, R_Y_, R_Z_) are:

36 $${\mathrm{R}}_{\mathrm{X}}=0$$

37 $${\mathrm{R}}_{\mathrm{Y}}=\sqrt{\sum_{m=1}^{3}{\left({o}_{m}+\left(\frac{\sum_{n=1}^{3}\left({r}_{n}-{o}_{n}\right)\left({a}_{n}-{o}_{n}\right)}{\sum_{n=1}^{3}{\left({a}_{n}-{o}_{n}\right)}^{2}}\right)\left({a}_{m}-{o}_{m}\right)-{r}_{m}\right)}^{2}}$$

38 $${\mathrm{R}}_{\mathrm{Z}}=\sqrt{\sum_{m=1}^{3}{\left({o}_{m}-{r}_{m}\right)}^{2}-\sum_{m=1}^{3}{\left({o}_{m}+\left(\frac{\sum_{n=1}^{3}\left({r}_{n}-{o}_{n}\right)\left({a}_{n}-{o}_{n}\right)}{\sum_{n=1}^{3}{\left({a}_{n}-{o}_{n}\right)}^{2}}\right)\left({a}_{m}-{o}_{m}\right)-{r}_{m}\right)}^{2}}$$

Four components of the lengthwise $\overrightarrow{\mathrm{OA}}$ and the crosswise vector $\overrightarrow{\mathrm{OR}}$ are:

39 $$\overrightarrow {{{\text{OA}}}}_{{\text{Y}}} = \cos \uptheta \cdot \sin \uppsi \cdot l$$

40 $$\overrightarrow {{{\text{OA}}}}_{{\text{Z}}} = \left( {\cos \upvarphi \cdot \cos \uppsi - \sin \upvarphi \cdot \sin \uptheta \cdot \sin \uppsi } \right) \cdot l$$

41 $$\begin{aligned} \overrightarrow {{{\text{OR}}}}_{{\text{Y}}} & = \cos \uptheta \cdot \cos \uppsi \cdot \sqrt {\mathop \sum \limits_{m = 1}^{3} \left( {o_{m} + \left( {\frac{{\mathop \sum \nolimits_{n = 1}^{3} \left( {r_{n} - o_{n} } \right)\left( {a_{n} - o_{n} } \right)}}{{\mathop \sum \nolimits_{n = 1}^{3} \left( {a_{n} - o_{n} } \right)^{2} }}} \right)\left( {a_{m} - o_{m} } \right) - r_{m} } \right)^{2} } \\ & \quad + \cos \uptheta \cdot \sin \uppsi \cdot \sqrt {\mathop \sum \limits_{m = 1}^{3} \left( {o_{m} - r_{m} } \right)^{2} - \mathop \sum \limits_{m = 1}^{3} \left( {o_{m} + \left( {\frac{{\mathop \sum \nolimits_{n = 1}^{3} \left( {r_{n} - o_{n} } \right)\left( {a_{n} - o_{n} } \right)}}{{\mathop \sum \nolimits_{n = 1}^{3} \left( {a_{n} - o_{n} } \right)^{2} }}} \right)\left( {a_{m} - o_{m} } \right) - r_{m} } \right)^{2} } \\ \end{aligned}$$

42 $$\begin{aligned} \overrightarrow {{{\text{OR}}}}_{{\text{Z}}} & = \left( {\cos \upvarphi \cdot \sin \uppsi - \sin \upvarphi \cdot \sin \uptheta \cdot \cos \uppsi } \right) \\ & \quad \cdot \sqrt {\mathop \sum \limits_{m = 1}^{3} \left( {o_{m} + \left( {\frac{{\mathop \sum \nolimits_{n = 1}^{3} \left( {r_{n} - o_{n} } \right)\left( {a_{n} - o_{n} } \right)}}{{\mathop \sum \nolimits_{n = 1}^{3} \left( {a_{n} - o_{n} } \right)^{2} }}} \right)\left( {a_{m} - o_{m} } \right) - r_{m} } \right)^{2} } \\ & \quad + \left( {\cos \upvarphi \cdot \cos \uppsi - \sin \upvarphi \cdot \sin \uptheta \cdot \sin \uppsi } \right) \\ & \quad \cdot \sqrt {\mathop \sum \limits_{m = 1}^{3} \left( {o_{m} - r_{m} } \right)^{2} - \mathop \sum \limits_{m = 1}^{3} \left( {o_{m} + \left( {\frac{{\mathop \sum \nolimits_{n = 1}^{3} \left( {r_{n} - o_{n} } \right)\left( {a_{n} - o_{n} } \right)}}{{\mathop \sum \nolimits_{n = 1}^{3} \left( {a_{n} - o_{n} } \right)^{2} }}} \right)\left( {a_{m} - o_{m} } \right) - r_{m} } \right)^{2} } \\ \end{aligned}$$

## Abbreviations

CT: Computerized tomography
XCP-ORA: EXtention Cone Paralleling-One Ring and Arm positioning system
PID: Portable indicating device
DXT: Dental X-ray tube
CAD/CAM: Computer-aided design/computer-aided manufacturing
CF: Correction factor
MBL: Marginal bone level
CMOS: Complementary metal oxide semiconductor
